# Supplementary material for: Patterns and drivers of recent disturbances across the temperate forest biome
Source: Nat Commun. 2018 Oct 19;9:4355. doi: 10.1038/s41467-018-06788-9 (PMC6195561; doi:10.1038/s41467-018-06788-9)
Supplement: Supplementary file 1 — Supplementary Information [file 41467_2018_6788_MOESM1_ESM.pdf]

Supplementary material

# Patterns and drivers of recent disturbances across the temperate forest biome

Andreas Sommerfeld, Cornelius Senf, Brian Buma, Anthony W. D'Amato, Tiphaine Després, Ignacio Díaz-Hormazabal, Shawn Fraver, Lee E. Frelich, Álvaro G. Gutiérrez, Sarah J. Hart, Brian J. Harvey, Hong S. He, Tomáš Hlásny, Andrés Holz, Thomas Kitzberger, Dominik Kulakowski, David Lindenmayer, Akira S. Mori, Jörg Müller, Juan Paritsis, George L. W. Perry, Scott L. Stephens, Miroslav Svoboda , Monica G. Turner, Thomas T. Veblen, Rupert Seidl

**Supplementary Figures**

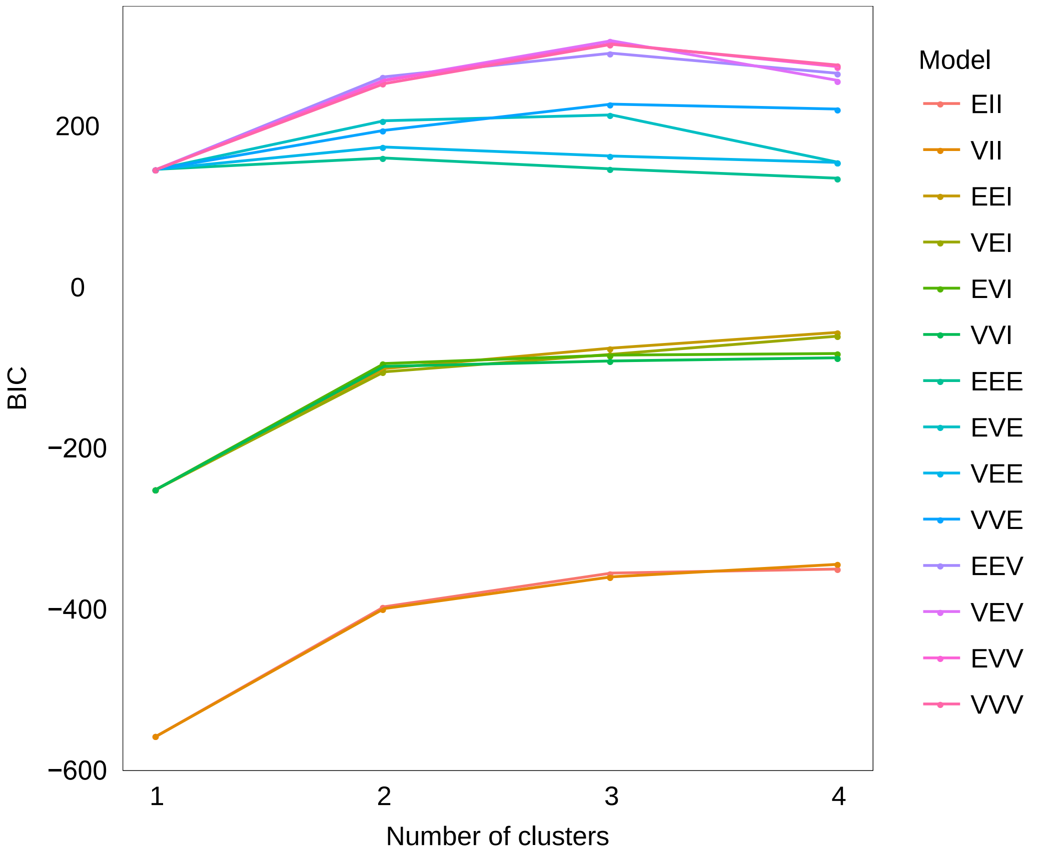


Supplementary Figure 1: Comparison of different clustering models (colors) and number of clusters using the Bayes Information Criterion (BIC). Models are identified using three letter codes describing the geometric characteristics of the approach, namely volume, shape, and orientation. E means equal and V means varying across components or clusters, while I refers to the identity matrix in specifying shape or orientation and is a special case of E. For details we refer to Fraley et al. [1].

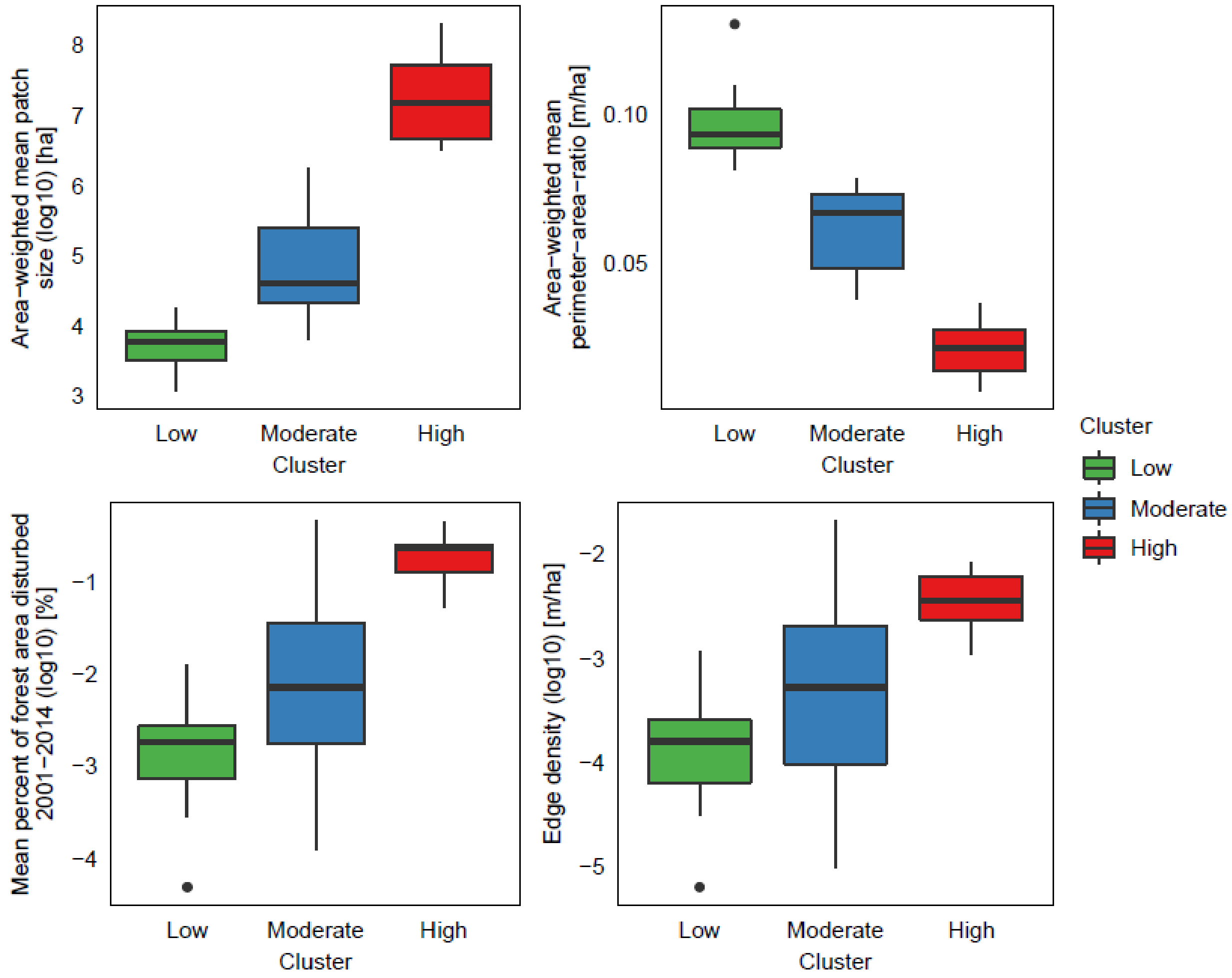


Supplementary Figure 2: Boxplots showing the distribution of the four disturbance metrics in the three clusters of disturbance activity. See also Table 1. Boxplots denote the median (center line) and interquartile range (box), with whiskers extending to three times the interquartile range and points indicating values outside this range. The number of experimental replicates equals the number of study sites per cluster (*Low:* 18, *Moderate:* 23, *High:* 9)

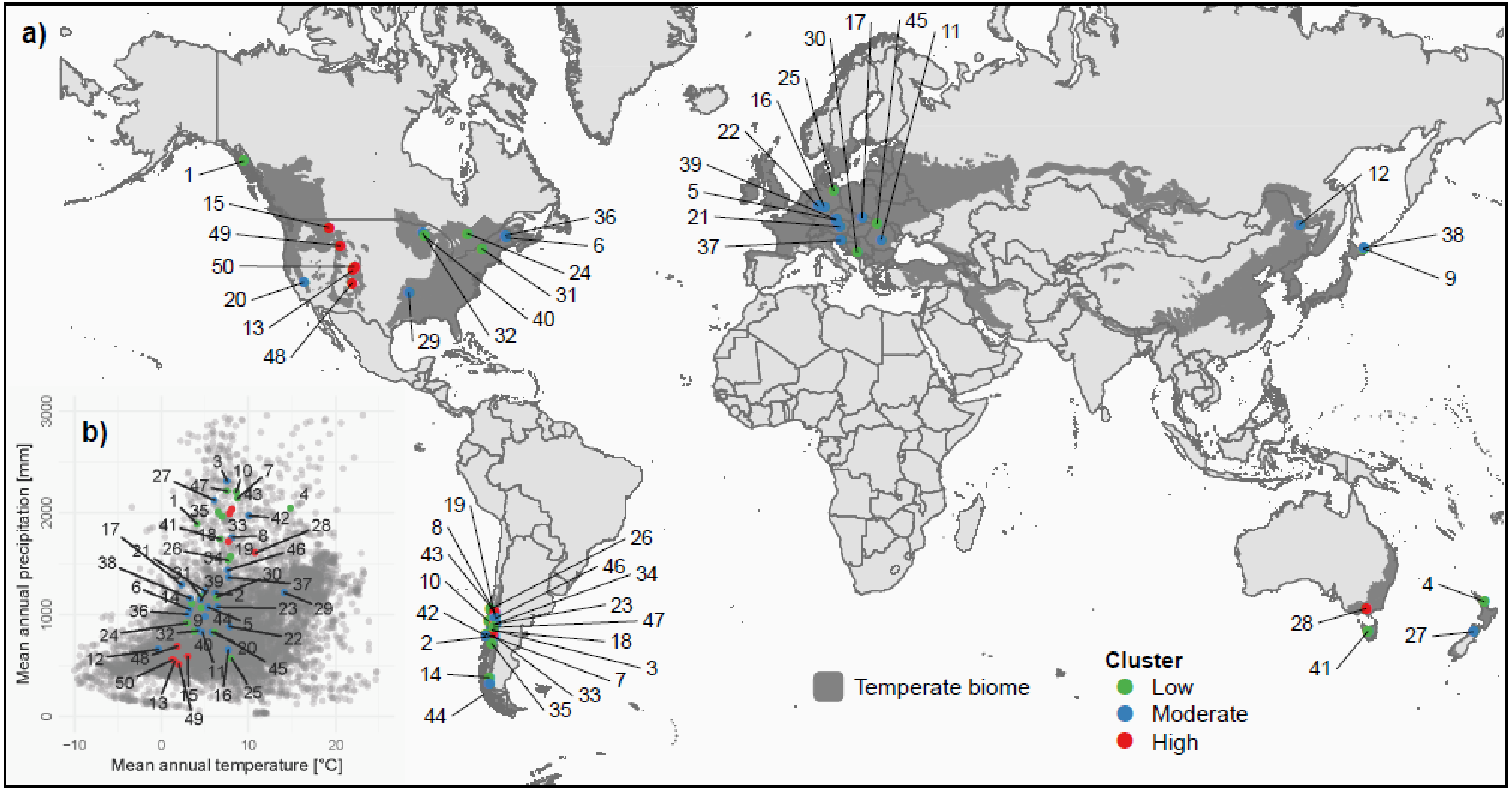


Supplementary Figure 3: A network of 50 protected landscapes across the temperate biome (biome area indicated in dark grey[2]). The panels show a) the geographic location, and b) the location of the landscapes in climate space. Study sites are colored according to their respective cluster, with green, blue and red representing clusters of *low*, *moderate* and *high* disturbance activity. Note that the climatic envelope of the biome (dark grey dots in panel b) is based on a sample of 10,000 4500 m × 4500 m grid cells throughout the biome.

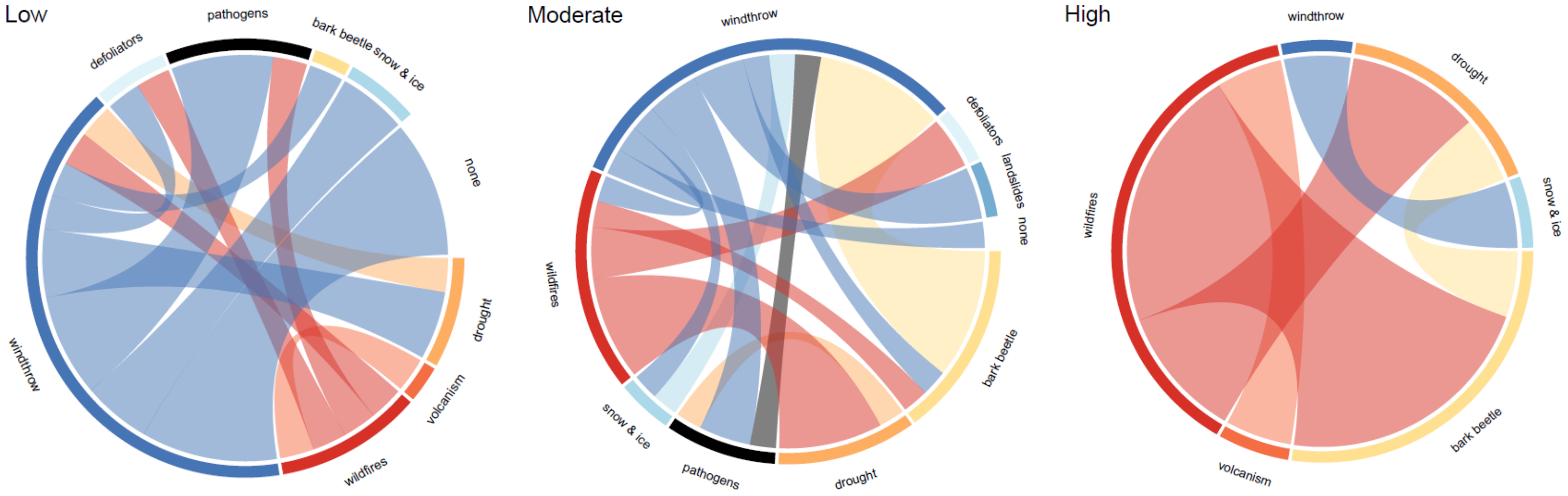


Supplementary Figure 4: Co-occurrences of the two most important disturbance agents per landscape in clusters with *low*, *moderate*, and *high* disturbance activity. The sector size in the outer circle indicates the relative prevalence of a disturbance agent in a respective cluster, while the links denote co-occurrence.

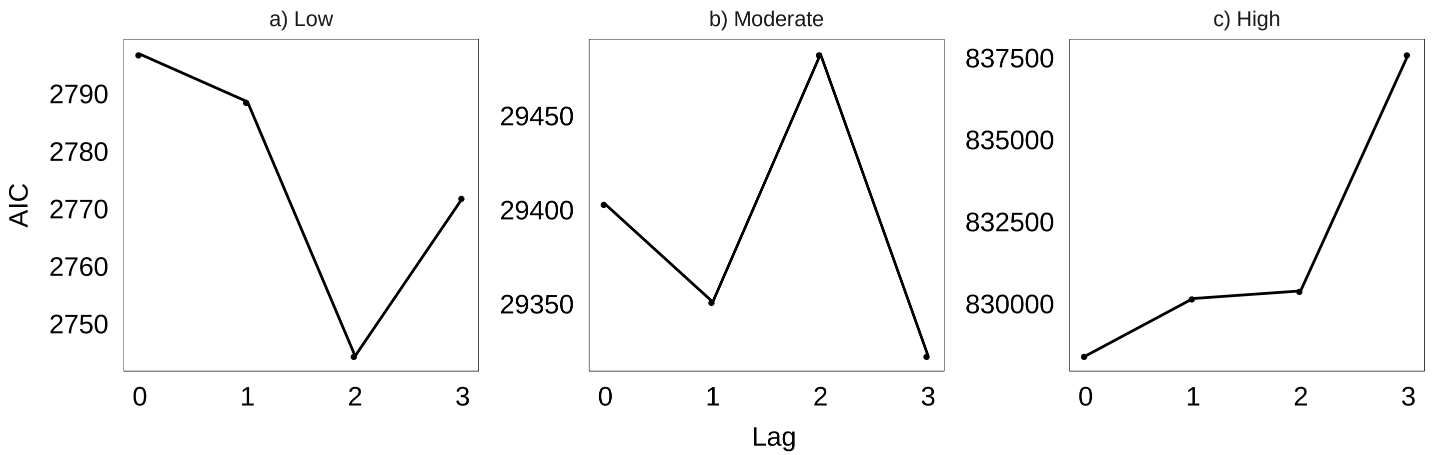


Supplementary Figure 5: Variable lag times (years) between climate anomaly and disturbance response, compared using the Akaike Information Criterion (AIC) of the respective GLMM. The lag with the lowest AIC was chosen in the final model.

**Supplementary Tables**

Supplementary Table 1: Summary of the 50 study sites. Disturbance characteristics (area-weighted mean patch size, area-weighted perimeter-area ratio, percent of area disturbed, and edge density) pertain to the years 2001 to 2014.

| # | Landscape name | Country | Local experts | Total area disturbed [ha] | Forest-cover [ha] | Main disturbance agents | Dominant tree species | Percent of area disturbed [%] | Edge density [m/ha] | Area-weighted mean patch size [ha] | Area-weighted perimeter-area ratio [m/ha] | Cluster |
|---|---|---|---|---|---|---|---|---|---|---|---|---|
| 1 | Admirality Island | USA | B. Buma | 192 | 367853 | windthrow, pathogens, snow & ice, landslides, lack of snow | *Tsuga heterophylla, Picea sitchensis, Tsuga mertensiana, Callitropsis nootkatensis, Pinus contorta* | 0.05 | 0.53 | 0.85 | 1011 | *Low* |
| 2 | Alerces | Argentina | A. Holz & J. Paritsis | 1338 | 154313 | wildfires, defoliators, snow & ice, drought | *Nothofagus pumilio, Nothofagus antarctica, Nothofagus dombeyi, Austrocedrus chilensis, Fitzroya cupressoides* | 0.87 | 6.53 | 4.39 | 754 | *Moderate* |
| 3 | Alerce Andino | Chile | Á. G. Gutiérrez & I. Díaz-Hormazábal | 38 | 36009 | windthrow, landslides | *Fitzroya cupressoides, Nothofagus betuloides, Nothofagus nitida, Nothofagus pumilio, Nothofagus dombeyi* | 0.11 | 0.71 | 2.07 | 670 | *Moderate* |
| 4 | Te Paparahi Conservation Area | New Zealand | G. L. W. Perry | 23 | 12378 | windthrow, herbivory, drought, landslides | *Kunzea robusta, Agathis australis, Beilschmiedia tarairi, Vitex lucens, Dysoxylum spectabile* | 0.19 | 1.55 | 1.15 | 821 | *Low* |
| 5 | Bavarian Forest | Germany | J. Müller | 1694 | 7271 | bark beetles, windthrow, pathogens | *Picea abies, Fagus sylvatica, Abies alba, Sorbus aucuparia, Acer pseudoplatanus* | 23.3 | 103.65 | 56.89 | 445 | *Moderate* |
| 6 | Baxter State Park | USA | S. Fraver | 169 | 69939 | windthrow, pathogens | *Picea rubens, Abies balsamea, Acer saccharum, Betula alleghaniensis, Fagus grandifolia* | 0.24 | 1.03 | 48.87 | 427 | *Moderate* |

| # | Landscape name | Country | Local experts | Total area disturbed [ha] | Forest-cover [ha] | Main disturbance agents | Dominant tree species | Percent of area disturbed [%] | Edge density [m/ha] | Area-weighted mean patch size [ha] | Area-weighted perimeter-area ratio [m/ha] | Cluster |
|---|---|---|---|---|---|---|---|---|---|---|---|---|
| 7 | Chiloé | Chile | Á. G. Gutiérrez & I. Díaz-Hormazábal | 2 | 34827 | windthrow | *Nothofagus nitida, Tepualia stipularis, Aextoxicon punctatum, Fitzroya cupressoides, Eucryphia cordifolia* | 0 | 0.06 | 0.12 | 1298 | *Low* |
| 8 | Conguillío | Chile | Á. G. Gutiérrez & I. Díaz-Hormazábal | 104 | 20885 | wildfires, drought, windthrow, snow & ice, landslides | *Nothofagus dombeyi, Nothofagus antarctica, Nothofagus pumilio, Araucaria araucana, Nothofagus alpina* | 0.5 | 2.93 | 4.07 | 587 | *Moderate* |
| 9 | Shiretoko Cool temperate | Japan | A. S. Mori | 9 | 7029 | windthrow, browsing, defoliators, snow & ice, landslides | *Picea jezoensis, Abies sachalinensis, Quercus crispula, Betula ermanii, Kalopanax septemlobus* | 0.13 | 1.06 | 0.62 | 810 | *Low* |
| 10 | Alerce Costero | Chile | Á. G. Gutiérrez & I. Díaz-Hormazábal | 15 | 22858 | windthrow | *Laureliopsis philippiana, Nothofagus nitida, Nothofagus dombeyi, Drimys winteri, Fitzroya cupressoides* | 0.06 | 0.55 | 0.57 | 860 | *Low* |
| 11 | Fagaras | Romania | M. Svoboda | 241 | 7736 | bark beetles, windthrow | *Fagus sylvatica, Abies alba, Picea abies* | 3.11 | 13.15 | 19.16 | 423 | *Moderate* |
| 12 | Feng Lin | China (PRC) | H. S. He | 243 | 17571 | wildfires, bark beetles, windthrow, defoliators, drought | *Pinus koraiensis, Abies nephrolepis, Betula costata, Picea koraiensis, Tilia amurensis* | 1.38 | 10.78 | 2.13 | 781 | *Moderate* |
| 13 | Flattops | USA | D. Kulakowski, T. T. Veblen & S. J. Hart | 5516 | 44540 | wildfires, bark beetles, drought | *Picea engelmannii, Abies lasiocarpa, Populus tremuloides, Pinus contorta, Pseudotsuga menziesii* | 12.38 | 17.16 | 1511.41 | 139 | *High* |

| # | Landscape name | Country | Local experts | Total area disturbed [ha] | Forest-cover [ha] | Main disturbance agents | Dominant tree species | Percent of area disturbed [%] | Edge density [m/ha] | Area-weighted mean patch size [ha] | Area-weighted perimeter-area ratio [m/ha] | Cluster |
|---|---|---|---|---|---|---|---|---|---|---|---|---|
| 14 | Glaciares | Argentina | A. Holz & J. Paritsis | 733 | 61828 | wildfires, defoliators, snow & ice (avalanches, ice storms, etc.) | *Nothofagus pumilio, Nothofagus antarctica, Nothofagus betuloides* | 1.19 | 11.66 | 0.65 | 984 | *Low* |
| 15 | Glacier | USA | B. J. Harvey & M. G. Turner | 153393 | 642334 | wildfires, bark beetles | *Pinus contorta, Pseudotsuga menziesii, Picea engelmannii, Abies lasiocarpa, Larix occidentalis* | 23.88 | 35.37 | 5882.1 | 148 | *High* |
| 16 | Hainich | Germany | J. Müller | 40 | 4458 | pathogens, windthrow, snow & ice | *Fagus sylvatica, Acer pseudoplatanus, Fraxinus excelsior, Quercus robur, Carpinus betulus* | 0.91 | 6.47 | 2.6 | 714 | *Moderate* |
| 17 | High Tatra | Slovakia | T. Hlasny | 388 | 4695 | bark beetles, windthrow, pathogens | *Pinus mugo, Picea abies, Sorbus aucuparia, Larix decidua, Betula sp* | 8.26 | 31.53 | 80.48 | 382 | *Moderate* |
| 18 | Hornopirén | Chile | Á. G. Gutiérrez & I. Díaz-Hormazábal | 23 | 19961 | windthrow, snow & ice | *Nothofagus betuloides, Nothofagus pumilio, Nothofagus nitida, Fitzroya cupressoides, Nothofagus dombeyi* | 0.12 | 1.17 | 0.41 | 1016 | *Low* |
| 19 | Huerquehue | Chile | Á. G. Gutiérrez & I. Díaz-Hormazábal | 12 | 9527 | drought, windthrow, snow & ice, landslides | *Nothofagus dombeyi, Nothofagus pumilio, Nothofagus obliqua, Nothofagus antarctica, Araucaria araucana* | 0.13 | 1.18 | 0.76 | 930 | *Low* |
| 20 | Illilouette Creek/ Yosemite | USA | S. L. Stephens | 1903 | 23221 | wildfires, drought, bark beetles, pathogens | *Abies concolor, Abies magnifica, Pinus contorta, Pinus jeffreyi* | 8.19 | 33.19 | 175.8 | 405 | *Moderate* |

| # | Landscape name | Country | Local experts | Total area disturbed [ha] | Forest-cover [ha] | Main disturbance agents | Dominant tree species | Percent of area disturbed [%] | Edge density [m/ha] | Area-weighted mean patch size [ha] | Area-weighted perimeter-area ratio [m/ha] | Cluster |
|---|---|---|---|---|---|---|---|---|---|---|---|---|
| 21 | Kalkalpen | Austria | R. Seidl | 492 | 13465 | windthrow, bark beetles, snow & ice, wildfires | *Picea abies, Fagus sylvatica, Larix decidua, Abies alba, Acer pseudoplatanus* | 3.65 | 19.32 | 4.6 | 529 | *Moderate* |
| 22 | Kellerwald | Germany | J. Müller | 241 | 5048 | bark beetles, windthrow, snow & ice, drought, pathogens | *Fagus sylvatica, Picea abies, Quercus robur, Larix europaea, Pinus sylvestris* | 4.78 | 24.32 | 5.53 | 509 | *Moderate* |
| 23 | Nahuel Lanin | Argentina | T. Kitzberger | 4506 | 628938 | wildfires, drought, volcanism, defoliators, snow & ice | *Nothofagus pumilio, Nothofagus antarctica, Nothofagus dombeyi, Austrocedrus chilensis, others* | 0.72 | 5.24 | 33.15 | 731 | *Moderate* |
| 24 | Malakisis | Canada | T.Després | 8 | 2861 | windthrow, bark beetles | *Acer saccharum, Betula alleghaniensis, Tsuga canadensis, Abies balsamea, Betula papyrifera* | 0.28 | 2.5 | 0.39 | 902 | *Low* |
| 25 | Müritz | Germany | J. Müller | 51 | 4077 | wildfires, windthrow | *Pinus sylvestris, Fagus sylvatica, Betula pendula, Alnus glutinosa, Quercus robur* | 1.26 | 11.43 | 0.87 | 906 | *Low* |
| 26 | Nahuelbuta | Chile | Á. G. Gutiérrez & I. Díaz-Hormazábal | 26 | 6065 | windthrow, drought, pathogens | *Araucaria araucana, Nothofagus dombeyi, Nothofagus obliqua, Nothofagus antarctica* | 0.43 | 3.81 | 1.36 | 882 | *Low* |

| # | Landscape name | Country | Local experts | Total area disturbed [ha] | Forest-cover [ha] | Main disturbance agents | Dominant tree species | Percent of area disturbed [%] | Edge density [m/ha] | Area-weighted mean patch size [ha] | Area-weighted perimeter-area ratio [m/ha] | Cluster |
|---|---|---|---|---|---|---|---|---|---|---|---|---|
| 27 | Nelson Lakes National Park | New Zealand | G. L. W. Perry | 204 | 52503 | windthrow, landslides, herbivory | *Lophonzonia menziesii, Fuscaspora fuscaspora, Fuscaspora cliffortioides, others* | 0.39 | 2.33 | 3.93 | 598 | *Moderate* |
| 28 | O'Shannassy | Australia | D. Lindenmayer | 5845 | 13241 | wildfires, drought | *Eucalyptus regnans, Eucalyptus delegatensis, Eucalyptus nitens, Eucalyptus obliqua, others* | 44.14 | 60.32 | 5304.76 | 137 | *High* |
| 29 | Ozarks | USA | H. S. He | 9 | 7264 | drought, pathogens, bark beetles, defoliators, snow & ice | *Quercus alba, Pinus echinata, Quercus rubra, Quercus veltutina, Carya tomentosa* | 0.12 | 0.86 | 2.2 | 722 | *Moderate* |
| 30 | Perućica | Bosnia and Herzegowina | M. Svoboda | 3 | 1408 | windthrow, pathogens, landslides, snow & ice, bark beetles | *Fagus sylvatica, Abies alba, Picea abies* | 0.23 | 2.26 | 0.28 | 981 | *Low* |
| 31 | Five Ponds | USA | A. D'Amato | 6 | 12574 | windthrow, pathogens, defoliators, snow & ice, drought | *Acer saccharum, Fagus grandifolia, Picea rubens, Tsuga canadensis, Betula alleghaniensis* | 0.05 | 0.51 | 0.27 | 1071 | *Low* |
| 32 | Porcupine | USA | L. E. Frelich | 30 | 24006 | windthrow, wildfires, defoliators, drought | *Acer saccharum, Tsuga canadensis, Betula alleghaniensis, Tilia americana, Acer rubrum* | 0.12 | 0.9 | 3.14 | 732 | *Moderate* |
| 33 | Pumalín | Chile | Á. G. Gutiérrez & I. Díaz-Hormazábal | 8728 | 169220 | windthrow, snow & ice | *Nothofagus nitida, Nothofagus dombeyi, Nothofagus pumilio, Nothofagus betuloides, Laureliopsis philippiana* | 5.16 | 11.07 | 4659.35 | 215 | *High* |

| # | Landscape name | Country | Local experts | Total area disturbed [ha] | Forest-cover [ha] | Main disturbance agents | Dominant tree species | Percent of area disturbed [%] | Edge density [m/ha] | Area-weighted mean patch size [ha] | Area-weighted perimeter-area ratio [m/ha] | Cluster |
|---|---|---|---|---|---|---|---|---|---|---|---|---|
| 34 | Puyehue | Chile | Á. G. Gutiérrez & I. Díaz-Hormazábal | 21531 | 67012 | volcanism, wildfires, windthrow, snow & ice | *Nothofagus pumilio, Nothofagus dombeyi, Laureliopsis philippiana, Nothofagus betuloides, Nothofagus antarctica* | 32.13 | 23.35 | 20045.21 | 73 | *High* |
| 35 | Queulat | Chile | Á. G. Gutiérrez & I. Díaz-Hormazábal | 82 | 11082 | windthrow, snow & ice | *Nothofagus betuloides, Nothofagus pumilio, Nothofagus dombeyi, Nothofagus nitida, Laureliopsis philippiana* | 0.74 | 6.93 | 0.68 | 932 | *Low* |
| 36 | Big Reed | USA | S. Fraver | 6 | 1929 | windthrow, pathogens | *Picea rubens, Abies balsamea, Thuja occidentalis, Acer saccharum, Fagus gradifolia* | 0.32 | 2.43 | 0.69 | 754 | *Moderate* |
| 37 | Risnjak | Croatia | M. Svoboda | 4 | 3224 | snow & ice, windthrow, bark beetles | *Fagus sylvatica, Abies alba, Picea abies* | 0.12 | 0.89 | 0.65 | 744 | *Moderate* |
| 38 | Shiretoko subalpine | Japan | A. S. Mori | 6 | 44069 | windthrow, snow & ice, defoliators, browsing, landslides | *Betula ermanii, Sorbus commixta, Sorbus matsumurana, Alnus maximowiczii, Acer tschonoskii* | 0.01 | 0.1 | 1.67 | 774 | *Moderate* |
| 39 | Sumava | Czech | M. Svoboda | 1095 | 2422 | bark beetles, windthrow | *Picea abies, Sorbus aucuparia* | 45.22 | 207.68 | 89.04 | 459 | *Moderate* |
| 40 | Sylvania | USA | L. E. Frelich | 11 | 6006 | windthrow, drought, defoliators | *Tsuga canadensis, Acer saccharum, Betula alleghaniensis, Tilia americana, Acer rubrum* | 0.18 | 1.63 | 0.51 | 929 | *Low* |

| # | Landscape name | Country | Local experts | Total area disturbed [ha] | Forest-cover [ha] | Main disturbance agents | Dominant tree species | Percent of area disturbed [%] | Edge density [m/ha] | Area-weighted mean patch size [ha] | Area-weighted perimeter-area ratio [m/ha] | Cluster |
|---|---|---|---|---|---|---|---|---|---|---|---|---|
| 41 | Tasmania | Australia | A. Holz | 13 | 45078 | wildfires, pathogens, drought | *Athrotaxis cupressoides, Eucalyptus coccifera* | 0.03 | 0.31 | 0.24 | 1096 | *Low* |
| 42 | Tepuhueico | Chile | Á. G. Gutiérrez & I. Díaz-Hormazábal | 72 | 19779 | windthrow | *Tepualia stipularis, Nothofagus nitida, Drymis winteri, Amomyrtus luma, Aextoxicon punctatum* | 0.37 | 2.63 | 2.11 | 719 | *Moderate* |
| 43 | Tolhuaca | Chile | Á. G. Gutiérrez & I. Díaz-Hormazábal | 668 | 4169 | wildfires, drought, windthrow, snow & ice, landslides | *Nothofagus obliqua, Nothofagus dombeyi, Nothofagus alpina, Nothofagus pumilio, Araucaria araucana* | 16.02 | 44.2 | 407.81 | 276 | *High* |
| 44 | Torres del Paine | Chile | A. Holz & J. Paritsis | 1456 | 43475 | wildfires, defoliators, snow & ice (avalanches, ice storms, etc.) | *Nothofagus pumilio, Nothofagus antarctica, Nothofagus betuloides* | 3.35 | 21.6 | 12.1 | 645 | *Moderate* |
| 45 | Uholka | Ukraine | M. Svoboda | 14 | 5489 | windthrow | *Fagus sylvatica, others* | 0.25 | 2.6 | 0.3 | 1024 | *Low* |
| 46 | Villarrica | Chile | Á. G. Gutiérrez & I. Díaz-Hormazábal | 19 | 24145 | wildfires, drought, windthrow, snow & ice, landslides | *Nothofagus pumilio, Nothofagus dombeyi, Nothofagus antarctica, Nothofagus obliqua, Araucaria araucana* | 0.08 | 0.54 | 1.71 | 695 | *Moderate* |
| 47 | Vicente Pérez Rosales | Chile | Á. G. Gutiérrez & I. Díaz-Hormazábal | 365 | 158085 | volcanism, wildfires, windthrow, snow & ice | *Nothofagus dombeyi, Nothofagus pumilio, Nothofagus betuloides, Nothofagus nitida, Eucryphia cordifolia* | 0.23 | 1.92 | 1.75 | 831 | *Low* |

| # | Landscape name | Country | Local experts | Total area disturbed [ha] | Forest-cover [ha] | Main disturbance agents | Dominant tree species | Percent of area disturbed [%] | Edge density [m/ha] | Area-weighted mean patch size [ha] | Area-weighted perimeter-area ratio [m/ha] | Cluster |
|---|---|---|---|---|---|---|---|---|---|---|---|---|
| 48 | Weminuche | USA | T. T. Veblen & S. J. Hart | 17666 | 77444 | bark beetles, drought, wildfires, defoliators | *Picea engelmannii, Abies lasiocarpa, Pinus contorta, Pinus flexilis, Populus tremuloides* | 22.81 | 82.84 | 316.74 | 363 | *High* |
| 49 | Yellowstone | USA | B. J. Harvey & M. G. Turner | 113924 | 915221 | wildfires, bark beetles, defoliators, pathogens | *Pinus contorta, Pseudotsuga menziesii, Picea engelmannii, Abies lasiocarpa, Pinus albicaulis* | 12.45 | 30.55 | 1469.1 | 245 | *High* |
| 50 | Mount Zirkel | USA | D. Kulakowski & B. Buma | 7953 | 32392 | wildfires, bark beetles | *Picea engelmanii, Abies latifolia, Populus tremuloides, Pinus contorta, Pseudotsuga menziesii* | 24.55 | 84.12 | 462.86 | 343 | *High* |

Supplementary Table 2: Multiple comparisons of species composition and weighted mean trait values among clusters of disturbance activity. Test statistics and p-values are based on approximate Kruskal-Wallis tests with 9,999 permutations. P-values were adjusted for multiple comparisons using false discovery rate (FDR) correction.

| Trait | Comparison of clusters | Statistic (H) | p-value | p-value (FDR-corrected) |
|---|---|---:|---:|---:|
| Dominance | Low – Moderate | 0.0699 | 0.7936 | 0.7936 |
| | Low – High | 2.9120 | 0.0880 | 0.1320 |
| | Moderate – High | 5.9930 | 0.0133 | 0.0399 |
| Conifers | Low – Moderate | 1.1780 | 0.2809 | 0.5320 |
| | Low – High | 0.9316 | 0.3547 | 0.5320 |
| | Moderate – High | 0.3500 | 0.5722 | 0.5722 |
| Height | Low – Moderate | 1.7950 | 0.1879 | 0.1879 |
| | Low – High | 5.8440 | 0.0147 | 0.0441 |
| | Moderate – High | 2.2140 | 0.1476 | 0.1879 |
| Wood density | Low – Moderate | 0.0994 | 0.7645 | 0.7645 |
| | Low – High | 5.5980 | 0.0154 | 0.0231 |
| | Moderate - High | 6.6440 | 0.0079 | 0.0231 |

Supplementary Table 3: Comparison of area-weighted mean patch size and area-weighted mean perimeter-area-ratio inside and outside of protected areas. Test statistics and p-values are based on approximate Kruskal-Wallis tests with 9,999 permutations.

| Metric | Cluster | Statistic (H) | p-value |
|---|---|---|---|
| Area-weighted mean patch size | Low | 26.2703 | < 0.0001 |
| | Moderate | 12.5885 | 0.0002 |
| | High | 0.0020 | 1.0000 |
| Area-weighted mean perimeter-area-ratio | Low | 24.9922 | < 0.0001 |
| | Moderate | 12.7448 | 0.0005 |
| | High | 0.5634 | 0.4777 |

Supplementary Table 4: Summary of the GLMMs testing the effect of temperature and precipitation anomalies as well as relative topographic ruggedness on disturbance probability. Values in parentheses are standard errors estimated from the GLMMs. Asterisks indicate the level of significance (*** P < 0.001, ** P < 0.01, * P < 0.05). Results of likelihood-ratio-tests comparing the full model to a spatial-only and a Null-model are also indicated.

| Cluster | Model | Intercept | Topographic Ruggedness Index | Precipitation anomaly | Temperature anomaly | Precipitation × temperature anomaly | AIC | Log-likelihood | $\chi^2$ (DF) | *P* |
|---|---|---|---|---|---|---|---|---|---|---|
| Low | Full | 0.02 (±0.36) | 0.30 (±0.04)*** | 0.33 (±0.07)*** | -0.20 (±0.07)** | -0.04 (±0.06) | 2744.36 | -1365.18 | 63.07 (3) | < 0.001 |
| | Spatial-only | 0.08 (±0.35) | 0.29 (±0.04)*** | - | - | - | 2801.42 | -1396.71 | 47.67 (1) | < 0.001 |
| | Null | 0.08 (±0.35) | - | - | - | - | 2847.09 | -1420.55 | - | - |
| Moderate | Full | -0.50 (±0.50) | -0.05 (±0.01)*** | -0.11 (±0.02)*** | -0.17 (±0.03)*** | -0.03 (±0.02)*** | 29322.28 | -14654.14 | 255.94 (3) | < 0.001 |
| | Spatial-only | -0.41 (±0.50) | -0.05 (±0.01)*** | - | - | - | 29572.22 | -14782.11 | 12.36 (1) | < 0.001 |
| | Null | -0.41 (±0.50) | - | - | - | - | 29582.58 | -14788.29 | - | - |
| High | Full | -0.65 (±0.24)** | -0.10 (±0.00)*** | -0.09 (±0.01)*** | 0.59 (±0.01)*** | -0.43 (±0.00)*** | 828401.56 | -414193.78 | 18073.93 (3) | < 0.001 |
| | Spatial-only | 0.07 (0.28) | -0.11 (±0.00)*** | - | - | - | 846469.48 | -423230.74 | 1670.36 (1) | < 0.001 |
| | Null | 0.07 (0.28) | - | - | - | - | 848137.85 | -424065.92 | - | |

Supplementary Table 5: Questionnaire to compile expert-based information on tree species, disturbance agents, and potential human interventions for the 50 protected landscapes. See Table S1 for the names of the local experts contacted.

| # | Question |
|---|---|
| 1 | What are the five most important tree species (in terms of basal area share) occurring on the landscape? Please give scientific names and approximate percentages. |
| 2 | List the major disturbance agents that were active on the landscape during 2001-2014 (in decreasing order of importance). |
| 3 | Which tree species where affected by these disturbance agents? |
| 4 | Were any human interventions undertaken during or after disturbance (e.g., fire suppression, salvage logging)? If so, please list them in decreasing order of their intensity. |

Supplementary Table 6: Known direct human interventions within the studied protected landscapes (see Supplementary Table 1 for more details on landscapes).

| # | Landscape name | Country | Main disturbance agents | Direct human interventions |
|---|---|---|---|---|
| 2 | Alerces | Argentina | wildfires, defoliators, snow & ice, drought | fire suppression |
| 8 | Conguillio | Chile | wildfires, drought, windthrow, snow & ice, landslides | fire suppression, irrigation |
| 11 | Fargaras | Romania | bark beetles, windthrow | sanitary cuts, salvage logging |
| 12 | Feng Lin | China (PRC) | wildfires, bark beetles, windthrow, defoliators, drought | fire & defoliator suppression, salvage logging |
| 13 | Flattops | USA | wildfires, bark beetles, drought | fire suppression |
| 14 | Glaciares | Argentina | wildfires, defoliators, snow & ice | fire suppression |
| 23 | Nahuel Lanin | Argentina | wildfires, drought, volcanism, defoliators, snow & ice | fire suppression |
| 28 | O'Shannassy | Australia | wildfires, drought | fire suppression |
| 29 | Ozarks | USA | drought, pathogens, bark beetles, defoliators, snow & ice | pathogen & defoliator suppression |
| 32 | Porcupine | USA | windthrow, wildfires, defoliators, drought | fire suppression |
| 34 | Puyehue | Chile | volcanism, wildfires, windthrow, snow & ice | fire suppression |
| 37 | Risnjak | Croatia | snow & ice, windthrow, bark beetles | sanitary cuts, salvage logging |
| 39 | Sumava | Czech | bark beetles, windthrow | sanitary cuts, salvage logging |
| 43 | Tolhuaca | Chile | wildfires, drought, windthrow, snow & ice, landslides | fire suppression, irrigation |
| 44 | Torres del Paine | Chile | wildfires, defoliators, snow & ice | fire suppression |
| 46 | Villarrica | Chile | wildfires, drought, windthrow, snow & ice, landslides | fire suppression, irrigation |
| 48 | Weminuche | USA | bark beetles, drought, wildfires, defoliators | fire suppression, salvage logging |

**Supplementary References**


1. Fraley, C., Raftery & Adrian, E. Model-based Methods of Classification : Using the mclust Software in Chemometrics. *J. Stat. Softw.* **18,** 1–13 (2007).
2. Olson, D. M. *et al.* Terrestrial ecoregions of the world: A new map of life on earth A new global map of terrestrial ecoregions provides an innovative tool for conserving biodiversity. *Bioscience* **51,** 933–938 (2001).
